# Sustained underweight in rural areas and emergence of overweight in urban Ethiopian women: a multivariate analysis of EDHS data 2000–2016

**DOI:** 10.1038/s41598-024-66409-y

**Published:** 2024-07-19

**Authors:** Amare Abera Tareke, Addis Alem, Wondwossen Debebe, Taddese Alemu Zerfu

**Affiliations:** 1 Department of Biomedical Sciences, College of Medicine and Health Sciences, Wollo University, Dessie, Ethiopia; 2 Department of Public Health, College of Medicine and Health Sciences, Wollo University, Dessie, Ethiopia; 3 International Food Policy Research Institute (IFPRI), Addis Ababa, Ethiopia; 4 College of Medicine and Health Sciences, Dilla University, Dilla, Ethiopia

**Keywords:** Body mass index, Overweight, Trend, Underweight, Women, Malnutrition, Obesity

## Abstract

A growing body of evidence indicates the emergence of overweight/obesity in developing countries before the battle against undernutrition has been won. We conducted this study to quantify the reduction of underweight and the emergence of overweight among Ethiopian women from 2000 to 2016 and evaluate factors explaining the progress. We used the four Ethiopian Demographic and Health Surveys (2000–2016) to analyze body mass index (BMI) trends among women. Data from 43,815 non-pregnant, non-puerperal reproductive-age women was used to evaluate the linear change in BMI and changes in the percentage of overweight and underweight over time. Using multivariate decomposition analysis of change in underweight and overweight percentages, we identified sources of change in BMI in the past 16 years of the survey periods. The BMI of Ethiopian reproductive-age women increased by 0.88 kg/m^2^ from 2000 to 2016. The increment was pronounced in urban areas with 1.46 kg/m^2^. There has been a significant reduction in underweight women since 2000 (p-value < 0.001), and 87.62% of the changes were attributed to behavioral changes toward weight management. And there was a significant upswing in overweight women from 2000 to 2016 (p-value < 0.001) as well. A compositional change of factors including region, women’s age, women’s educational status, religion, type of place of residence, and use of contraceptives contributed to 57.51% of the observed increment in the percentage of overweight women. A relatively slow decrease in underweight and an increment in overweight have been observed. This progress can be disaggregated into persistent underweight in the rural and poorest, and swift development of overweight in the urban and richest communities. Targeted nutrition interventions for both underweight and overweight women are mandatory. Nutritional interventions in Ethiopia should focus on behavioral change to reduce hunger and malnutrition as well as to avert the emergence of overweight or obesity in the affected communities.

## Introduction

Globally, the prevalence of underweight remains high, and the burden of overweight/obesity is rising in many regions of the world^1^. A steady, high body mass index (BMI) was observed in developed countries, while a rise in BMI was evident in many developing regions^2^. BMI, with or without other variables, can provide reliable evidence in the prediction of overall mortality^3^.

BMI and overall mortality have a U-shaped relationship. Mortality is lowest among those with a BMI of 25–27.4 kg/m^2^. Women with a BMI < 22.5 kg/m^2^ had significantly higher overall mortality. Additionally, women with a BMI < 20 kg/m^2^ displayed an increased risk of cardiovascular mortality^4^. High BMI is frequently linked with non-communicable diseases (NCDs). Overweight and obesity are major risk factors for a number of chronic diseases, including cardiovascular diseases such as heart disease and stroke, which are leading causes of death worldwide^5^. There is a rising concern over higher BMI which led to the inclusion of adiposity as global NCD target^6,7^. Not only higher BMI, the slimmest (BMI < 18 kg/m^2^), although had a lower risk for cardiovascular disorders, the overall mortality was higher^8^.

The two extremes of BMI, which are addressed as a double burden of malnutrition in much literature, impose a great deal of problems at individual, societal, and intergenerational levels, especially in women^9^. The emerging dual burden of malnutrition impacts the health outcomes of mothers and offspring related to childbirth in lower- and middle-income countries. For example, in India, persistently short adult stature and adult obesity are associated with increased cesarean delivery^10^. Furthermore, evidence is compelling on the metabolic complications of the double burden of malnutrition. The recognized cardio-metabolic risks of being overweight are exacerbated among stunted mothers^11^.

In Africa, an analysis of prevalence and time trends in overweight and obesity in women in 24 countries indicated an increasing trend in overweight and obesity in all countries in the last three decades^12^. The prevalence doubled or tripled in half of the 24 countries in urban areas. Addressing the consequences of underweight or undernutrition is the primary agenda for African countries. While battling the burden of undernutrition, these countries are facing the other edge of the sword, overweight/obesity.

In Ethiopian women, there is growing evidence indicating the emergence of overweight/obesity especially in urban areas^13,14^. A recent cross-sectional study in southern Ethiopia gave us a glimpse of the coming uncertainty, that, more than a third of the women were either overweight or obese^14^. In the capital (Addis Ababa) a steeper rise in urban women’s obesity was recorded from successive demographic and health surveys^13^.

As BMI is the current metric for anthropometric characteristics in adults, the knowledge of population-based trends has several advantages^15^. Besides the common interpretation of BMI as indicator of an individual’s fatness, it is widely used as a risk factor for morbidity and mortality^16^ and is widely used in formulating public health policies. The current study tried to show the progress Ethiopia has made in managing undernutrition and the neglected fact that we are facing as a developing country: the burden of overweight and obesity. In this study, we determined the trend in BMI from four Ethiopian Demographics and Health Survey data and the factors responsible for the change during the same period. The result of the current study also indicated the progress of underweight and overweight among Ethiopian women. Due to the paucity of such data, this study will provide concrete input for the current nutritional intervention strategies.

## Methods

### Study design and setting

Ethiopia is the second-most populous country in Africa next to Nigeria with a population of more than one hundred million. Administratively, Ethiopia is divided into eleven geographical regions (Tigray, Afar, Amhara, Oromia, Somali, Benishangul-Gumuz, SNNPR, Gambella, and Harari, as well as newly added Sidama and South West Ethiopia regions) and two administrative cities, Addis Ababa and Dire Dawa. Ethiopia shares the boundaries in the North with Eritrea, in the South with Kenya and Somalia, in the West with South Sudan and North Sudan, and in the East with Djibouti and Somalia.

The current study uses the four Ethiopian Demographics and Health Surveys (EDHS) conducted in 2000, 2005, 2011, and 2016. These surveys were conducted with nationally representative samples from all regions of the country. Details of sampling design, including sampling framework and sample implementation, and response rates are provided on respective EDHS reports (http://www.measuredhs.com). The four EDHS data were used to determine the level and trend in BMI in reproductive-age women. These four DHS data were used to identify factors responsible for the change and to indicate the shift in variables.

### Data source

The dataset used in this study was obtained from the MEASURE DHS database at http://dhsprogram.com/data/ after getting the approval letter from the DHS program office. The EDHSs conducted from 2000 to 2016 have a population of 61,635 reproductive-age women. For the current study, we used 43,815 (sampling weights applied) women excluding pregnant mothers, mothers at puerperium, and unmeasured BMI. The survey was conducted with nationally representative samples from all of the country’s regions with multiple enumeration areas.

### Sampling procedures

The DHS utilizes a two-stage cluster sampling procedure; where a group of adjacent households serves as a primary sampling unit. In the first stage, a stratified sample of enumeration areas (EAs) was selected with probability proportional to size: in each stratum, a sample of a predetermined number of EAs is selected independently with probability proportional to the EA’s measure of size. In the selected EAs, a listing procedure was performed such that all dwellings/households were listed.

In the second stage, after a complete household listing is conducted in each of the selected EAs, a fixed (or variable) number of households is selected by equal probability systematic sampling in the selected EAs. In each selected household, a household questionnaire is completed to identify women aged 15–49. The detailed sampling procedure for DHS is available at https://dhsprogram.com/publications/publication-dhsm4-dhs-questionnaires-and-manuals.cfm.

For example: during 2016 EDHS, in the first stage, a total of 645 clusters (202 in urban areas and 443 in rural areas) were selected based on the 2007 population and housing census. A household listing was carried out in all of the selected clusters from September to December 2015 and served as a sampling frame for the selection of households in the second stage.

**Figure 1:**
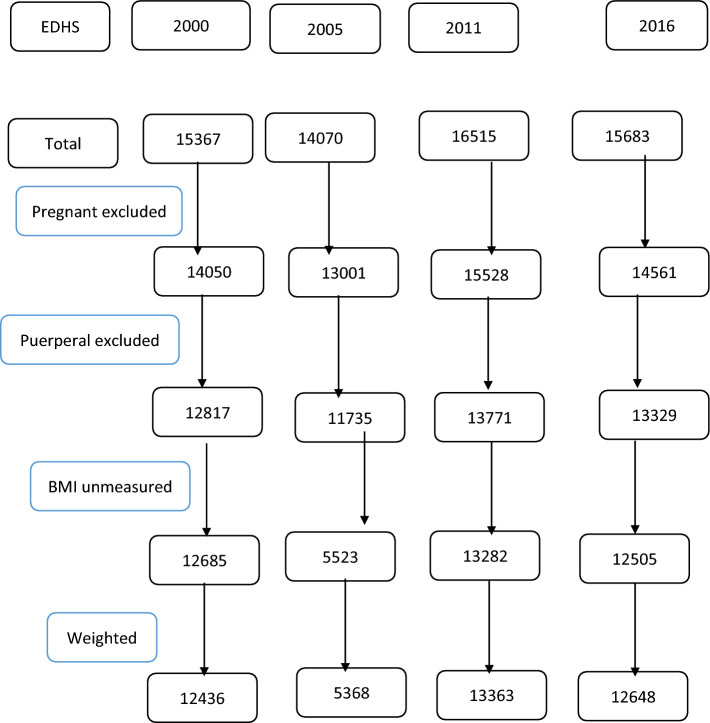
Schematic illustration of women included in the study. As indicated in the figure the four EDHSs recruited a total of 61,635 reproductive age women, pregnancy, puerperium, and mothers with unmeasured BMI were excluded. It is obvious that BMI is not a good indicator during pregnancy, besides, puerperal mothers were excluded from this study. In EDHS 2000, there were 1313 pregnant, 1233 lactating puerperal women and 132 missing BMI data. In 2005, 1069 pregnant, 1266 puerperal and 6212 women had un measured BMI; whereas in 2011 987 mothers were pregnant, 1757 were lactating, and 489 mothers didn’t measure BMI. In the recent (2016) EDHS, the number of pregnant mothers, puerperal and unmeasured BMI were 1122, 1232 and 824 respectively. A total of 43,815 reproductive age women (after applying sampling weights) were included for this study.

The representative samples of 18,008 households were selected for the 2016 EDHS. From the interviewed households, 16,583 eligible women were identified for individual interviews. Interviews were completed with 15,683 women, yielding a response rate of 95%^16^. In this study, a total of 43,996 mothers were included from the four EDHS datasets (EDHS 2000 = 12,685, EDHS 2005 = 5523, EDHS 2011 = 13,282, and EDHS 2016 = 12,505). The steps followed in identifying eligible mothers are summarized in Fig. 1.

### Study variables

The outcome variable of the study was a change in BMI across different times of the EDHS. BMI is calculated by dividing an individual’s weight in kilograms by an individual’s squared height in meters. The variable was classified as “underweight” (BMI < 18.5), “normal” (18.5 ≤ BMI < 25), overweight (25 ≤ BMI < 30), and “obesity” (BMI ≥ 30)^17^. However, for the sake of computational feasibility, the last two categories were merged as overweight.

Again, for the feasibility of our statistical analysis, multivariate nonlinear decomposition analysis with dichotomous outcome was applied. First we analyzed the change of underweight over time by comparing with normal and overweight individuals. In this case, we have dichotomous underweight versus others (normal and overweight merged). We also determined the change in overweight over time by comparing with the weights and the normal BMI. We have dichotomous outcomes (others vs overweight) in this case as well.

### Statistical analysis

The data were cleaned and analyzed using STATA Version 16.0. First, descriptive statistics were performed to summarize the background characteristics of women after applying women’s individual sampling weights. Second, the trend of BMI among total women, rural as well as urban women from 2000 to 2016 was computed after applying sampling weights. Percentage point differences were calculated to indicate positive and negative changes over different EDHS phases.

The study used Multivariate Decomposition Analysis to investigate changes in BMI among women. Multivariate Decomposition Analysis is an effective tool for comprehending the origins of disparities and shifts in outcomes among different groups or over time. By separating the contributions of various factors and breaking down observed differences in outcomes, such as BMI, between two groups or time periods, Multivariate Decomposition Analysis provides valuable insights for policymakers to tackle inequalities and enhance outcomes.

Our analysis considered both changes in population composition (endowment) and population behavior (coefficient) in relation to BMI. The endowment effect refers to the portion of the difference attributable to variations in explanatory variables, while the coefficient effect pertains to the differing impact of these explanatory variables across groups. Through the application of Multivariate Decomposition Analysis, the study identified and quantified these effects, offering a clearer understanding of the factors driving changes in BMI among women.

In the present case, Multivariate Decomposition Analysis was used to identify sources of change in BMI in the past 16 years of the survey periods. Decomposing the overall gap into these components, helps to understand the relative contribution of each factor. Decomposition analysis are used to understanding differences in nutritional and health outcomes that can shed light on whether observed gaps are due to starting point differences (endowments) or how effectively groups utilize their resources (coefficients)^18^. If the endowments effect is dominant, policies might focus on equalizing opportunities or resources. Conversely, a large coefficients effect might suggest a need for targeted training or mentorship programs.

We used logit-based decomposition analysis to identify factors contributing to the change in BMI over time. The observed difference in BMI between different surveys is additively decomposed into a characteristic (E) component—variation in the population composition across the survey and a coefficient (C) component—change in the behavior of the population.

The observed difference was decomposed in two components. The first component was difference due to variation in population composition across the survey. The second was due to change in the behavior of the population. The change in the outcome was due to either change in population composition or change in behavior of the population. The model structure for decomposition analysis was:

$${\text{Logit }}\left( {\text{A}} \right) - {\text{Logit }}\left( {\text{B}} \right) \, = \, [\beta_{0A} - \beta_{0B} ] \, + \Sigma \beta_{ijA} \left[ {X_{ijA} - X_{ijB} } \right] \, + \, \Sigma X_{ijB} [\beta_{ijA} - \beta_{ijB} ],$$

*where, β*_*0A*_* is the intercept in the regression* equation for EDHS 2016, *β*_*0B*_* is* the intercept in the regression equation for EDHS 2000, *β*_*ijA*_* is the coefficient of the jth category of the ith determinant for EDHS 2016, β*_*ijB*_* is the coefficient of the jth category of the ith determinant of EDHS 2000, X*_*ijA*_* is the proportion of the jth category of the ith determinant of EDHS 2016, X*_*ijB*_* is the proportion of jth category of the ith determinant of EDHS 2000*.

### Ethical approval and consent to participate

Procedures and questionnaires for standard DHS surveys have been reviewed and approved by ICF Institutional Review Board (IRB). Additionally, country-specific DHS survey protocols are reviewed by the ICF IRB and typically by an IRB in the host country. ICF IRB ensures that the survey complies with the U.S. Department of Health and Human Services regulations for the protection of human subjects (45 CFR 46), while the host country IRB ensures that the survey complies with laws and norms of the nation (https://dhsprogram.com/Methodology/Protecting-the-Privacy-of-DHS-Survey-Respondents.cfm).

Our study doesn’t involve collection of information from subjects. We sent a one-page proposal abstract of the study to DHS program office. They gave a permission to access the data.

## Result

### Respondents description

A total of 43,815 reproductive-age women (after applying sampling weights) were included in this study. The number of respondents (weighted) per DHS was 1246, 5368, 13,362, and 12,648 from earlier to recent surveys respectively. Table 1 presents the characteristics of respondents in each of the four surveys. As indicated, the percentage of women with higher education increased from 0.63% in 2000 to 5.99% in 2016. Another notable change was the percentage of working women during the times of survey increased from 42.35 to 64.72% in 2016. On the other hand, the number of family members, total children ever born, and total births in the last 5 years of the surveys decreased from 2000 to 2016.

**Table 1:** Description of the respondents in EDHS 2000 to 2016.

Variables	EDHS 2000	EDHS 2005	EDHS 2011	EDHS 2016
	Freq (%)	Freq (%)	Freq (%)	Freq (%)
Region				
Tigray	805.82 (6.48)	356.20 (6.64)	940.66 (7.04)	936.60 (7.41)
Afar	146.02 (1.17)	56.17 (1.05)	113.49 (0.85)	97.65 (0.77)
Amhara	3110.45 (25.01)	1328.20 (24.76)	3788.60 (28.35)	3210.59 (25.38)
Oromia	4733.11 (38.06)	1841.14 (34.30)	4716.84 (35.30)	4376.11 (34.60)
Somali	132.75 (1.07)	179.57 (3.34)	213.03 (1.59)	313.22 (2.48)
Benishangul Gumz	124.37 (1.00)	47.57 (0.89)	136.47 (1.02)	122.99 (0.97)
SNNP	2616.41 (21.04)	1176.57 (21.92	2513.62 (18.81)	2638.47 (20.86)
Gambela	32.72 (0.26)	18.52 (0.35)	58.95 (0.44)	36.67 (0.29)
Harari	33.95 (0.27)	16.27 (0.30)	39.42 (0.29)	27.63 (0.22)
Addis Aba	632.48 (5.09)	318.26 (5.93)	787.41 (5.89)	816.48 (6.46)
Dire Dawa	68.23 (0.55)	29.058693 (0.54)	54.91 (0.41)	71.56 (0.57)
Head				
Male	9476.58 (76.20)	4083.21 (76.06)	9926.58 (74.28)	9388.39 (74.23)
Female	2959.70 (23.80)	1285.09 (23.94)	3436.83 (25.72)	3259.57 (25.77)
Age				
15–19	3352.82 (26.96)	1454.22 (27.09)	3622.13 (27.10)	2986.59 (23.61)
20–24	2110.33 (16.97)	896.86 (16.71)	2217.12 (16.59)	2044.71 (16.17)
25–29	1826.47 (14.69)	808.77 (15.07)	2270.78 (16.99)	2157.63 (17.06)
30–34	1331.99 (10.71)	607.52 (11.32)	1514.41 (11.33)	1788.18 (14.14)
35–39	1382.76 (11.12)	604.60 (11.26)	1486.79 (11.13)	1547.15 (12.23)
40–44	1216.24 (9.78)	497.21 (9.26)	1101.96 (8.25)	1149.92 (9.09)
45–49	1215.65 (9.78)	499.12 (9.30)	1150.20 (8.61)	973.78 (7.70)
Educational level				
No education	9126.49 (73.39)	3370.85 (62.79)	6474.72 (48.45)	5885.60 (46.53)
Primary	2070.00 (16.64)	1280.89 (23.86)	5251.99 (39.30)	4474.67 (35.38)
Secondary	1161.14 (9.34)	633.74 (11.81)	984.44 (7.37)	1530.17 (12.10)
Higher	78.65 (0.63)	82.81 (1.54)	652.25 (4.88)	757.52 (5.99)
Contraceptive method				
No method	11,568.46 (93.02)	4707.43 (87.69)	10,348.52 (77.44)	8979.52 (71.00)
Folkloric method	3.46 (0.03)		5.18 (0.04)	
Traditional	174.63 (1.40)	39.19 (0.73)	131.99 (0.99)	71.43 (0.56)
Modern method	689.74 (5.55)	621.67 (11.58)	2877.71 (21.53)	3597.02 (28.44)
Anemia level				
Severe	NA	52.74 (1.07)	60.09 (0.46)	70.66 (0.57)
Moderate	NA	333.30 (6.77)	294.99 (2.26)	511.48 (4.11)
Mild	NA	843.09 (17.13)	1652.10 (12.63)	2200.51 (17.67)
Not anemic	NA	3691.97 (75.02)	11,071.29 (84.65)	9670.53 (77.66)
Cigarette smoking				
No	NA	5357.49 (99.83)	13,339.52 (99.82)	12,566.08 (99.35)
Yes	NA	8.89 (0.17)	23.89 (0.18)	81.89 (0.65)
Marital status				
Never married	3627.82 (29.17)	1656.43 (30.86)	4280.25 (32.03)	3808.79 (30.11)
Currently married	7056.53 (56.74)	3035.01 (56.54)	7476.69 (55.95)	7524.50 (59.49)
Formerly married	1751.93 (14.09)	676.85 (12.61)	1606.47 (12.02)	1314.68 (10.39)
Partner's occupation				
Did not work	53.47 (0.61)	41.01 (1.11)	113.39 (1.26)	589.21 (7.83)
Prof., Tech., Manag.	293.19 (3.33)	118.12 (3.20)	418.82 (4.64)	383.57 (5.10)
Clerical	92.50 (1.05)	8.91 (0.24)	135.02 (1.50)	52.62 (0.70)
Sales	528.89 (6.02)	267.62 (7.25)	819.45 (9.08)	529.27 (7.03)
Agric-employee	6973.66 (79.31)	2992.50 (81.04)	6588.03 (72.96)	4623.30 (61.44)
Services	229.13 (2.61)	48.33 (1.31)	179.70 (1.99)	244.95 (3.26)
Skilled manual	452.19 (5.14)	121.36 (3.29)	663.06 (7.34)	552.54 (7.34)
Unskilled manual	148.16 (1.69)	80.29 (2.17)	112.05 (1.24)	218.23 (2.90)
Don't know	21.62 (0.25)	14.70 (0.40)		107.41 (1.43)
Others				223.40 (2.97)
Currently working				
No	5265.40 (42.35)	3674.96 (68.48)	8076.33 (60.48)	8185.65 (64.72)
Yes	5265.40 (42.35)	1691.14 (31.52)	5277.76 (39.52)	4462.32 (35.28)
Religion				
Orthodox	6365.80 (51.19)	2693.99 (50.18)	6705.22 (50.21)	5765.73 (45.59)
Muslin	3555.59 (28.59)	1527.59 (28.46)	3449.76 (25.83)	3611.67 (28.56)
Traditional	411.98 (3.31)	73.27 (1.36)	93.99 (0.70)	76.16 (0.60)
Catholic	146.06 (1.17)	83.52 (1.56)	136.52 (1.02)	95.49 (0.75)
Protestant	1924.47 (15.47)	955.35 (17.80)	2872.71 (21.51)	3042.90 (24.06)
Other	32.39 (0.26)	34.57 (0.64)	95.26 (0.71)	56.02 (0.44)
Current age	28.24 (0.09)	28.02 (0.13)	27.72 (0.08)	28.36 (0.09)
Household members	5.99 (0.02)	5.87 (0.03)	5.71 (0.02)	5.54 (0.02)
Children ever born	2.98 (0.02)	2.98 (0.04)	2.75 (0.02)	2.75 (0.02)
Births in last 5 years	0.64 (0.01)	0.65 (0.01)	0.57 (0.01)	0.57 (0.01)
BMI	19.74 (0 .02)	20.11 (0.04)	20.20 (0.03)	20.62 (0.03)

NA: data not available.

### Trends in BMI and urban–rural disparities

**Figure 2:**
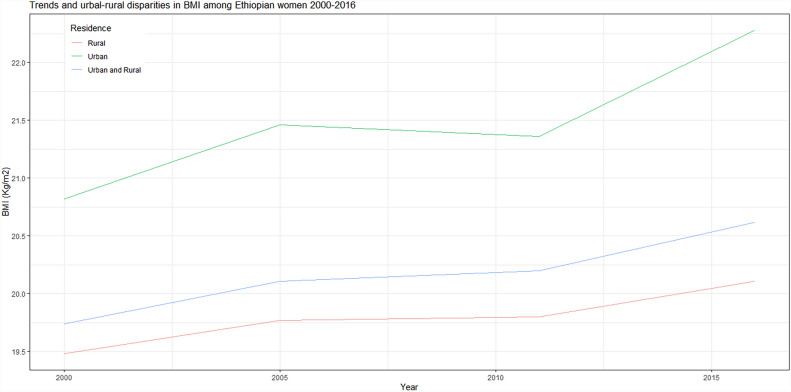
Mean BMI changes in Ethiopian women from 2000 to 2016. It also displayed mean BMI changes in urban and rural women. Women in the urban Ethiopia had higher mean BMI at 2000 than the rural women (20.82 kg/m^2^ and 19.48 kg/m^2^), and the changes towards 2016 were also higher in urban women.

The BMI of non-pregnant, non-puerperal Ethiopian reproductive-age women increased by 0.88 kg/m^2^ from 2000 to 2016. The increment for the total women was pronounced in urban dwellers with 1.46 kg/m^2^, while the rise in BMI in rural women was relatively slowly moving with 0.63 kg/m^2^. Trends in BMI, as well as urban–rural disparities, are given in Fig. 2.

**Figure 3:**
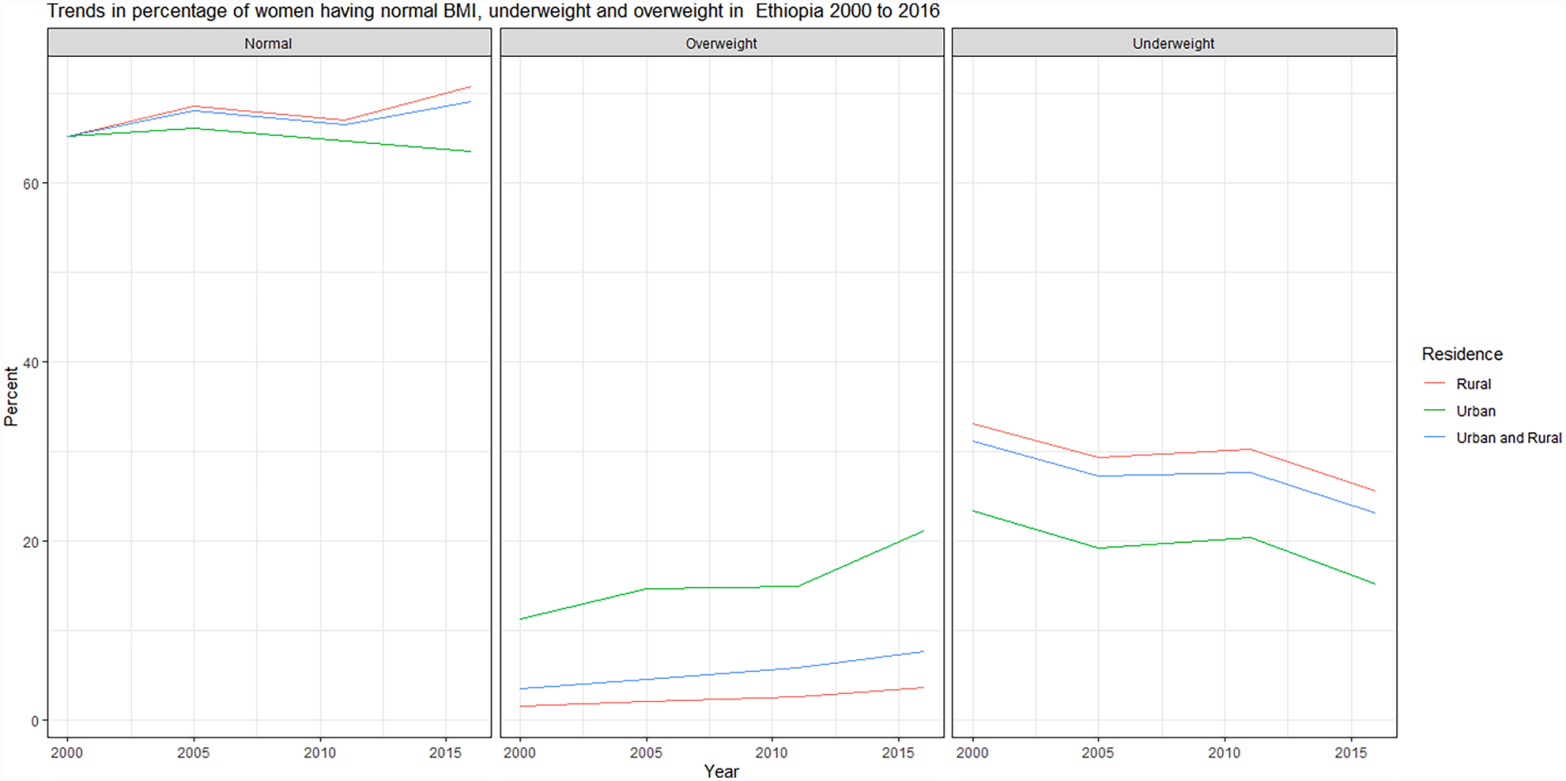
The trends in percentage of women having normal BMI, underweight and overweight in Ethiopia 2000 to 2016. The percentage of women underweight had decreased and overweight’s increased.

The percentage of underweight women has decreased, and the percentage of overweight women showed an increment, especially in urban areas. Table 2 and Fig. 3 indicate the urban–rural trends in BMI as classified by WHO. Of note is that the percentage of normal (BMI 18.5–24.9 kg/m^2^) has increased from 65.26% in 2000 to 69.08% in 2016 nationally. Again, nationally the percentage of underweights has decreased gradually, as the percentage of overweight women increased concomitantly.

**Table 2:** Trends in percentage of women underweight/overweight as classified by WHO, EDHS 2000 to 2016, urban–rural disparities.

	2000	2005	2011	2016
National				
Normal	65.26	68.1	66.47	69.08
Underweight	31.25	27.34	27.72	23.18
Overweight	3.49	4.56	5.81	7.74
Urban				
Normal	65.2	66.11	64.68	63.58
Underweight	23.43	19.24	20.38	15.24
Overweight	11.38	14.65	14.93	21.18
Rural				
Normal	65.28	68.59	67.09	70.76
Underweight	33.19	29.35	30.24	25.62
Overweight	1.54	2.05	2.68	3.63

Interestingly, in urban women, the percentage of “normal” dropped in 2016, from the 2000 figure (65.2% to 63.58%). This change is coupled with a decrease the underweights and increased overweight, signifying that the increment in overweight bypasses the decrement of underweights.

### Multivariate decomposition analysis

The percentage point differences in underweight and overweight women are given in Table 3. Generally, the table indicated the overall decrement in the percentage of underweight women and the increment in the percentage of overweight women. Regional variations exist in percentage changes; for example, little improvement has been observed in the reduction of underweight mothers in the Tigray region, whereas, the Benishangul Gumz region showed remarkable progress in a similar outcome (18.78 percentage points). The Amhara region registered the lowest percentage point increment in overweight women and Addis Ababa had the highest percentage point increment over the study period (12.83). As shown in Table 3, there was decrement in the percentage of overweight women who attended higher education, the percentage point difference was − 4.35.

**Table 3:** The percentage point differences in underweight and overweight Ethiopian women 2000 to 2016.

	Underweight			Overweight		
	2016	2000	Difference	2016	2000	Difference
Region						
Tigray	35.24	36.20	− 0.96	5.96	1.22	4.74
Afar	40.44	41.69	− 1.25	8.05	4.67	3.38
Amhara	23.64	32.80	− 9.16	3.48	1.84	1.64
Oromia	25.52	29.68	− 4.16	7.63	3.51	4.12
Somali	31.67	48.79	− 17.12	15.02	4.16	10.86
Ben-Gumz	20.75	39.53	− 18.78	7.68	2.01	5.67
SNNP	15.76	32.09	− 16.33	5.86	2.79	3.07
Gambela	31.43	39.90	− 8.47	8.72	1.06	7.66
Harari	21.49	25.37	− 3.88	20.49	10.38	10.11
Addis Aba	13.80	18.08	− 4.28	29.42	16.59	12.83
Dire Dawa	22.94	27.76	− 4.82	21.40	13.25	8.15
Sex of household head						
Male	23.67	31.86	− 8.19	6.73	2.87	3.86
Female	21.79	29.29	− 7.5	10.83	5.64	5.19
Age in 5-year groups						
15–19	29.42	38.85	− 9.43	3.66	2.19	1.47
20–24	21.15	23.96	− 2.81	5.58	3.30	2.28
25–29	18.90	25.16	− 6.26	8.10	4.06	4.04
30–34	19.92	24.83	− 4.91	10.75	3.95	6.8
35–39	22.60	30.66	− 8.06	10.72	4.94	5.78
40–44	22.68	31.92	− 9.24	11.86	4.90	6.96
45–49	25.33	39.10	− 13.77	9.45	3.36	6.09
Highest educational level of the respondent						
No education	24.18	32.24	− 8.06	4.85	2.11	2.74
Primary	24.54	31.07	− 6.53	7.42	4.60	2.82
Secondary	16.99	24.68	− 7.69	12.63	11.19	1.44
Higher	19.94	18.23	1.71	22.94	27.29	− 4.35
Current contraceptive method						
No method	24.91	31.82	− 6.91	7.25	2.91	4.34
Any method	18.95	23.62	− 4.67	9.09	11.72	− 2.63
Type of place of residence						
Urban	15.24	23.43	− 8.19	21.25	11.42	9.83
Rural	25.62	33.19	− 7.57	3.66	1.58	2.08
Number of household members						
< 5	22.13	28.74	− 6.61	8.85	3.67	5.18
≥ 5	23.77	32.35	− 8.58	7.20	3.47	3.73
Number of children 5 and under in household						
≤ 1	23.02	31.09	− 8.07	8.48	4.09	4.39
> 1	23.78	31.65	− 7.87	5.30	2.15	3.15
Total children ever born						
≤ 2	23.12	31.78	− 8.66	7.89	3.73	4.16
> 2	23.26	30.59	− 7.33	7.65	3.28	4.37
Births in last 5 years						
≤ 1	23.22	32.33	− 9.11	8.35	3.81	4.54
> 1	22.98	26.54	− 3.56	4.38	2.32	2.06
Religion						
Orthodox	22.84	30.29	− 7.45	8.97	3.87	5.1
Muslim	27.35	33.74	− 6.39	6.55	3.20	3.35
Others	19.17	30.16	− 10.99	7.06	3.14	3.92
Currently/formerly/never in union						
Never in union	25.53	35.89	− 10.36	6.02	3.71	2.31
Currently	22.09	29.15	− 7.06	8.03	3.10	4.93
Formerly	22.61	30.09	− 7.48	11.48	4.89	6.59
Respondent currently working						
No	24.99	33.95	− 8.96	5.52	3.76	1.76
Yes	19.87	29.27	− 9.4	11.95	3.36	8.59
Type of earnings from respondent's work						
Not paid	22.53	32.44	− 9.91	4.96	1.74	3.22
Cash only	17.67	25.93	− 8.26	15.35	6.30	9.05
Cash and in-kind	18.60	33.09	− 14.49	8.65	1.34	7.31
In-kind only	25.92	29.18	− 3.26	5.29	1.02	4.27

#### Underweight

There was a significant change in women underweight since 2000 (p-value < 0.001). As shown in Table 4, 87.62% of the change was attributed to the behavioral changes toward weight management controlling the effect of changes in compositional factors. Region had a statistically significant effect of coefficient contribution to the observed change in underweight. The Amhara and SNNP regions decreased the proportion of underweight women by 9.16 and 16.33 percentage points (Table 3), where behavioral change towards BMI plays a significant role for the two regions.

**Table 4:** Multivariate decomposition analysis of percentage changes in underweight among Ethiopian women 2000–2016.

Variables	Difference due to characteristics (E)		Difference due to coefficients (C)	
	Coefficient (95% CI)	Pct	Coefficient (95% CI)	pct
Region				
Tigray				
Afar	0.00001 (− 0.00035, 0.00038)	0.02	− 0.00005 (− 0.00116, 0.00106)	− 0.06
Amhara	0.00062 (0.00019, 0.00105)**	0.71	0.03281 (0.01563, 0.04999)**	37.84
Oromia	− 0.00293 (− 0.00510, − 0.00077)**	− 3.38	0.02188 (− 0.00277, 0.04652)	25.23
Somali	0.00062 (0.00006, 0.00117)	0.71	0.00151 (0.00074, 0.00229)**	1.74
Ben-Gumz	0.00006 (0.00001, 0.00010)*	0.07	0.00222 (0.00126, 0.00317)**	2.56
SNNP	− 0.00457 (− 0.00783, − 0.00130)**	− 5.27	0.03321 (0.01691, 0.04950)**	38.30
Gambela	0.00003 (− 0.00000, 0.00007)	0.04	0.00021 (0.00002, 0.00039)*	0.24
Harari	− 0.00006 (− 0.00011, − 0.00001)*	− 0.07	0.00021 (− 0.00004, 0.00047),	0.25
Addis Aba	0.00475 (0.00144, 0.00807)**	5.48	0.00128 (− 0.00240, 0.00495)	1.47
Dire Dawa	− 0.00006 (− 0.00011, − 0.00001)*	− 0.07	0.00026 (− 0.00026, 0.00078)	0.30
Sex of household head				
Male				
Female	− 0.00034 (− 0.00171, 0.00102)	− 0.40	− 0.00681 (− 0.02091, 0.00729)	− 7.85
Age in 5-year groups				
15–19				
20–24	− 0.00062 (− 0.00133, 0.00008)	− 0.72	− 0.00470 (− 0.01724, 0.00784)	− 5.42
25–29	0.00080 (− 0.00036, 0.00196)	0.93	− 0.00124 (− 0.01468, 0.01219)	− 1.43
30–34	0.00195 (− 0.00095, 0.00485)	2.25	0.00141 (− 0.00909, 0.01192)	1.63
35–39	0.00073 (− 0.00039, 0.00185)	0.85	0.00665 (− 0.00501, 0.01832)	7.67
40–44	− 0.00037 (− 0.00110, 0.00037)	− 0.42	0.00752 (− 0.00413, 0.01918)	8.68
45–49	− 0.00080 (− 0.00247, 0.00087)	− 0.92	0.01075 (− 0.00088, 0.02237)	12.40
Highest educational level of the respondent				
No education				
Primary	− 0.00303 (− 0.00736, 0.00131)	− 3.49	− 0.00467 (− 0.01364, 0.00429)	− 5.39
Secondary	0.00046 (− 0.00147, 0.00239)	0.53	0.00132 (− 0.00483, 0.00748)	1.53
Higher	− 0.00320 (− 0.00717, 0.00076)	− 3.70	− 0.00059 (− 0.00215, 0.00098)	− 0.68
Current contraceptive method				
No method				
Any method	0.00240 (− 0.00485, 0.00966)	2.77	0.00011 (− 0.00521, 0.00543)	0.12
Type of place of residence				
Urban				
Rural	0.00850 (0.00144, 0.01557)*	9.80	− 0.05814 (− 0.11933, 0.00305)	− 67.05
Number of household members				
< 5				
≥ 5	− 0.00018 (− 0.00239, 0.00204)	− 0.20	0.00875 (− 0.02627, 0.04376)	10.09
Number of children 5 and under in household				
≤ 1				
> 1	− 0.00069 (− 0.00402, 0.00263)	− 0.80	0.00972 (− 0.00837, 0.02780)	11.20
Total children ever born				
≤ 2				
> 2	0.00068 (− 0.00059, 0.00195)	0.79	− 0.02005 (− 0.05508, 0.01499)	− 23.12
Births in last 5 years				
≤ 1				
> 1	0.00076 (− 0.00233, 0.00385)	0.88	− 0.00532 (− 0.02033, 0.00969)	− 6.14
Religion				
Orthodox				
Muslim	0.00022 (− 0.00023, 0.00067)	0.25	− 0.00017 (− 0.01488, 0.01455)	− 0.19
Others	0.00010 (− 0.00089, 0.00110)	0.12	− 0.00188 ( − 0.01634, 0.01259)	− 2.16
Currently/formerly/never in union				
Never				
Currently	0.00012 (− 0.00004, 0.00029)	0.14	− 0.00772 (− 0.05007, 0.03464)	− 8.90
Formerly	− 0.00060 (− 0.00173, 0.00053)	− 0.69	− 0.00352 (− 0.01760, 0.01057)	− 4.06
Respondent currently working				
No				
Yes	0.00373 (− 0.00364, 0.01110)	4.30	− 0.03459 (− 0.08643, 0.01725)	− 39.89
Type of earnings from respondent's work				
Not paid				
Cash only	0.00039 (− 0.00054, 0.00132)	0.45	− 0.00457 (− 0.02414, 0.01500)	− 5.27
Cash and in-kind	0.00057 (− 0.00052, 0.00166)	0.66	0.00205 (− 0.00221, 0.00631)	2.36
In-kind only	0.00133 (− 0.00327, 0.00593)	1.53	− 0.00993 (− 0.02312, 0.00326)	− 11.45
Overall	0.01074 (− 0.00932, 0.03080)	12.38	0.07598 (0.04709, 0.10487)**	87.62

CI; confidence interval, pct; percentage *p value ≤ 0.05, **p value ≤ 0.01.

As indicated in the table the change in the population composition contribute to 12.38% of the change in underweight. Region and place of residence were significant factors in this category. On the other hand, after controlling the effect of compositional factors 87.62% of the change in underweight was due to the effects of characteristics.

Compositional factors contributed to 12.38% of the observed change in underweight percentage among Ethiopian women. Covariates in this category involve region and type of place of residence. As shown in Table 2, the percentage of underweight women dropped from 31.25 to 23.18% in 2016, this observed change was attributed to the compositional change in place of residence and other significant variables (shown in Table 4).

#### Overweight

There was a significant increase in women overweight from 2000 to 2016 (p value < 0.001) in Ethiopia. Table 5 depicts the contribution of both compositional (Endowment) and behavioral (coefficient) factors to the rise of overweight. Compositional change of factors contributed to 57.51% of the observed increment in the percentage of overweight women. Region, women’s age, women’s educational status, religion, type of place of residence, and use of contraceptive were among the significant covariates for the compositional change observed in overweight mothers.

**Table 5:** Multivariate decomposition analysis of percentage changes in overweight among Ethiopian women 2000–2016.

Variables	Difference due to characteristics (E)		Difference due to coefficients (C)	
	Coefficient (95% CI)	Pct	Coefficient (95% CI)	pct
Region				
Tigray				
Afar	0.00054 (0.00014, 0.00094)**	− 0.77	0.00039 (− 0.00012, 0.00091)	0.56
Amhara	0.00017 (− 0.00011, 0.00045)	− 0.24	0.01171 (− 0.00113, 0.02454)	− 16.65
Oromia	0.00272 (0.00134, 0.00410)**	− 3.87	0.00400 (− 0.00863, 0.01663)	− 5.68
Somali	− 0.00132 (− 0.00179, − 0.00084)**	1.87	− 0.00022 (− 0.00058, 0.00014)	0.31
Ben-Gumz	− 0.00004 (− 0.00007, − 0.00002)**	0.06	0.00011 (− 0.00049, 0.00070)	− 0.15
SNNP	0.00168 (0.00005, 0.00332)*	− 2.39	0.00291 (− 0.00578, 0.01159)	− 4.13
Gambela	− 0.00004 (− 0.00008, − 0.00001)*	0.06	− 0.00012 (− 0.00025, 0.00002)	0.17
Harari	0.00006 (0.00003, 0.00009**	− 0.09	0.00002 (− 0.00008, 0.00012)	− 0.03
Addis Aba	− 0.00347 (− 0.00508, − 0.00186)**	4.93	0.00076 (− 0.00069, 0.00221)	1.08
Dire Dawa	0.00006 (0.00003, 0.00009)**	− 0.09	0.00009 (− 0.00012, 0.00030)	− 0.13
Sex of household head				
Male				
Female	0.00059 (− 0.00077, 0.00195)	− 0.84	0.00658 (0.00035, 0.01280)*	− 9.35
Age in 5-year groups				
15–19				
20–24	0.00032 (− 0.00039, 0.00103)	− 0.46	0.00295 (− 0.00375, 0.00966)	− 4.20
25–29	− 0.00123 (− 0.00238, − 0.00009)*	1.76	0.00259 (− 0.00359, 0.00877)	− 3.68
30–34	− 0.00516 (− 0.00787, − 0.00246)**	7.34	− 0.00103 (− 0.00599, 0.00392)	1.47
35–39	− 0.00190 (− 0.00291, − 0.00089)**	2.70	− 0.00003 (− 0.00530, 0.00525)	0.04
40–44	0.00149 (0.00080, 0.00217)**	− 2.12	0.00135 (− 0.00365, 0.00635)	− 1.92
45–49	0.00279 (0.00123, 0.00434)**	− 3.96	− 0.00035 (− 0.00543, 0.00473)	0.50
Highest educational level of the respondent				
No education				
Primary	− 0.00870 (− 0.01399, − 0.00340)**	12.37	− 0.00194 (− 0.00618, 0.00230)	2.76
Secondary	− 0.00336 (− 0.00502, − 0.00170) **	4.78	− 0.00107 (− 0.00321, 0.00107)	1.52
Higher	− 0.00765 (− 0.01092, − 0.00439)**	10.88	0.00003 (− 0.00033, 0.00039)	− 0.04
Current contraceptive method				
No method				
Any method	− 0.00736 (− 0.01359, − 0.00114)*	10.47	0.00075 (− 0.00098, 0.00247)	− 1.06
Type of place of residence				
Urban				
Rural	− 0.01286 (− 0.01769, − 0.00802)**	18.28	0.00305 (− 0.01891, 0.02501)	− 4.34
Number of household members				
< 5				
≥ 5	0.00016 (− 0.00219, 0.00252)	− 0.23	0.00959 (− 0.00569, 0.02488)	− 13.64
Number of children 5 and under in household				
≤ 1				
> 1	0.00205 (− 0.00203, 0.00612)	− 2.91	− 0.00688 (− 0.01636, 0.00260)	9.78
Total children ever born				
≤ 2				
> 2	− 0.00005 (− 0.00121, 0.00111)	0.07	− 0.00822 (− 0.02197, 0.00554)	11.68
Births in last 5 years				
≤ 1				
> 1	− 0.00355 (− 0.00714, 0.00004)	5.05	0.00366 (− 0.00418, 0.01149)	− 5.20
Religion				
Orthodox				
Muslim	− 0.00043 (− 0.00092, 0.00005)	0.61	0.00470 (− 0.00127, 0.01067)	− 6.69
Others	0.00095 (− 0.00000, 0.00191)*	− 1.35	0.00450 (− 0.00099, 0.00999)	− 6.40
Currently/formerly/never in union				
Never in union				
Currently	− 0.00005 (− 0.00020, 0.00011)	0.07	− 0.00830 (− 0.02627, 0.00968)	11.80
Formerly	0.00125 (− 0.00001, 0.00251)	− 1.78	− 0.00364 (− 0.00924, 0.00196)	5.17
Respondent currently working				
No				
Yes	0.00217 (− 0.00547, 0.00980)	− 3.08	0.00041 (− 0.02456, 0.02538)	− 0.59
Type of earnings from respondent's work				
Not paid				
Cash only	− 0.00069 (− 0.00171, 0.00032)	0.98	0.00266 (− 0.00610, 0.01141)	− 3.78
Cash and in-kind	− 0.00006 (− 0.00112, 0.00100),	0.09	− 0.00065 (− 0.00342, 0.00212)	0.93
In-kind only	0.00271 (− 0.00398, 0.00939)	− 3.85	− 0.00226 (− 0.01043, 0.00591)	3.21
Overall	0.04044 (0.04808, 0.03281)**	57.51	− 0.02988 (− 0.04105, − 0.01871)**	42.49

CI; confidence interval, pct; percentage *p value ≤ 0.05, **p value ≤ 0.01.

This table depicts decomposition changes of overweight among reproductive age women in Ethiopia. As shown, keeping the effects of coefficients variation in the population composition across surveys contributed to 57.51% of the change in overweight. These variables are region, women’s age, women’s educational status, religion, type of place of residence, and use of contraceptive.

As shown in Table 5, keeping the compositional factors constant, 42.49% of the change in the percentage of overweight women was attributed to behavioral changes toward BMI. These factors include the sex of the household head. As shown in Table 3, female-headed households had a speedy drive towards overweight-5.19% than male-headed households-3.86%.

## Discussion

In 16 years of the survey period, Ethiopian women increased 0.88 kg/m^2^ of BMI. This figure was significant and the urban women contributed much to the national BMI rise, which was 1.47 kg/m^2^. The percentage of underweight women dropped from 31.25% in 2000 to 23.18% in 2016, during this time the percentage of overweight/obese women increased from 3.49 to 7.74%. Despite the decreased percentage of underweight women in urban areas of Ethiopia, the number of women having normal BMI decreased from 65.2% in 2000 to 63.58% in 2016, signifying that the drop in the percentage of underweight was overwhelmed by the accelerated development of overweight.

Global trends regarding BMI indicated the age-standardized mean BMI increased from 22.1 kg/m^2^ in 1975 to 24.4 kg/m^2^ in 2014 among women^19^. During this period global prevalence of underweight decreased from 14.6 to 9.7% and the prevalence of overweight increased from 6.4 to 14.9%^19^. This trend has also been observed in lower- and middle-income countries since the 2000s. Most of such countries decreased the prevalence of underweight, while countries like Senegal, Madagascar, and Mali failed to do so, and the prevalence in the three countries was increasing^20^. While this trend of increased overweight and decreased underweight was consistent across sub-Saharan African countries^12,21^, the ratio of underweight to overweight can be used as a proxy to measure countries’ progress in reducing underweight while maintaining a stable percentage of overweight. In the Ethiopian context, the ratio of underweight to overweight decreased from 8.95 in 2000 to 2.99 in 2016 nationally. This is achieved by decreasing underweight and increasing overweight, and in fact, underpins the decrement of underweight was faster. In urban women, the ratio decreased from 2.06 in 2000 to 0.72 in 2016, which indicates the development of overweight was accelerating while 15.24% of the women are still underweight. In most developing countries, there is an emergence of overweight/obesity before the battle against underweight has been won, as observed in Ethiopia^22^.

Our finding disclosed that geographical endowment differences (regional differences or place of residence) contributed to the reduction in prevalence of underweight. Urban women are more likely to be overweight, and rural women are more likely to be underweight. In rural areas, women mostly engaged in agricultural activities, which involves physical exertion and therefore unlikely to gain as much weight as the urban women. Moreover, women in rural areas are less exposed to the western lifestyle and the nutritional transition in the urban areas is not likely to be prevalent in a rural setting^23^. Furthermore, it is likely that whereas urban women have more than enough to eat, women in rural have less.

Women’s age contributed to the increase in prevalence of overweight. Body composition does not remain the same throughout life. Reduction of muscle mass begins in the 30s’ and concomitant increase in adipose tissue will take place^24^. In addition various hormonal changes like ovarian steroids, growth hormone, insulin (impaired B cell function) coupled with decreased physical activity in a later age, all push towards overweight and obesity^24^.

Modern contraceptive use is associated with increased prevalence of overweight. Since most contraceptives are synthetic steroids, they tend to cause fat deposition in a different part of the body leading to overweight and obesit^25^.

Although, behavioral factors play the lion’s share in significantly reducing the percentage of underweight, both compositional and behavioral factors were responsible for the increment of overweight over the past 16 years. While the current study showed behavioral factors had stronger effect on the change in underweight, a study among adolescent girls in Ethiopia pointed out compositional changes were major drivers of change for reduction of thinness^26^. These changes could related to “modernization” including increasing GDP per capita, urbanization, and women’s empowerment^27^. Findings from other countries indicated both the compositional and behavioral factors were important elements as drivers change for overweight and obesity^28^.

To address both underweight and overweight in Ethiopian women, multisectoral approach, promoting diversified diet including animal source foods for underweights, promote healthy snacks and meals, reduce the consumption of soda, diversified diet including fruits and vegetables for overweight, and implementation of nutrition education are important strategies^29^.

This study used nationally representative samples of the country from DHS. The DHS data is worldwide data implemented across various countries and at multiple points in time within a country. It provides comparability, consistency, and the best-quality survey results. It strengthens the quality of data through probability sampling, full coverage of the target population, use of suitable sample size and household listing, and pre-selection of households^30^.

Besides, this study showed the trend of both underweight and overweight at the same time. In countries like Ethiopia, where malnutrition is a two-edged sword, the analysis of both under and over-nutrition was critical. Sub-Saharan Africa is experiencing the double burden of malnutrition; high levels of undernutrition on one side and a growing burden of overweight/obesity on the other side^31^. Undernourishment in sub-Saharan Africa increased between 2010 and 2016^32^. Meanwhile, overweight/obesity is increasing in all age groups, with girls and women being more affected than boys and men^32^. The introduction of cheap processed foods and non-alcoholic beverages into African markets drives the consumption of unhealthy diets^31^. Besides, sedentary lifestyle has role in the development of obesity^33^. Progress toward the goal of ending hunger and malnutrition by 2030 requires intensified efforts to reduce undernutrition and focused action on the reduction of obesity and diet-related NCDs^31^.

For statistical feasibility, we dichotomize BMI^34^. First, to evaluate underweight, normal BMI and overweight was merged. And in the second scenario, the outcome variable (y = 1), if BMI ≥ 25 kg/m^2^, and y = 0 otherwise. Associated with this procedure, although, obesity was not such a burning issue in Ethiopia; the study didn’t show the change in obesity. Equally, to its strength of showing underweight and overweight trends; (1) the overweight category was added to the obese, and (2) the study didn’t report decomposition analysis on the obesity category. We ran the analysis to understand the status of obesity in Ethiopian women, and there was an increment from 2000 to 2016 (shown in the supplementary material, Supplementary Tables 1 and 2). In 2000 1.3% of the women were obese, this figure increased to 3.9% in 2016 nationally. The increment in the proportion of obese women was largely attributed to the urban women, where, urban obesity increased from 4.57 to 12.6% in the same period. Little change has been observed in rural women regarding obesity. Similar trends in obesity have been observed, in several sub-Saharan African countries^35^.

One critical issue in this analysis was the failure to analyze BMI changes based on the wealth index. This was raised from the unavailability of wealth index in 2000 EDHS data, but, is present in subsequent reports. We analyzed BMI trends and the contribution of wealth index from 2005 to 2016 (given in the supplementary material, supplementary Table 3). The mean BMI of the poorest was 19.52 kg/m^2^ in 2005 and decreased to 19.39 kg/m^2^ in 2016. Generally, the poor, the middle, and the rich had shown an increment in BMI, the increment of BMI in the rich quantile was greater than poor and the middle (the two were comparable). As expected, the richest had the highest increase in BMI-which was 1.01 kg/m^2^ in the decade. Surprisingly, the mean BMI ratio of the richest/the poorest was 1.09 in 2005, this ratio increased to 1.16 in 2016. It was a significant compositional factor for decrement in underweight in the richest quartile, and an increase in overweight in the rich and richest quartiles from 2005 to 2016, given in the supplementary material (supplementary Tables 8 and 9).

In developing countries both individual and country-level wealth were important factors^36,37^. Individual-level wealth index and country-level average national income were associated with increased BMI. The BMI of the wealthiest quantile was increasing fast compared with the low and stable poorest quantile. This signifies the poorest stay thinner and the richest grow fatter. The association between BMI and wealth was positive in many developing countries was not affected by survey periods, and remained so recently^37^.

## Conclusion

The percentage of underweight women decreased from 2000 to 2016. The decrement was pronounced in urban areas. Meanwhile, there was increment of overweight women nationally. Generally, the progress can be dis-aggregated as persistent underweight in rural and the poorest, and emergence of overweight in the urban and richest. Change in the population composition across surveys contributed to the increment of overweight. Behavioral changes largely contributed to the reduction of underweight. Nutritional interventions in Ethiopia should focus on the reduction of underweight in rural areas and lowest wealth quantile, and weight management in urban and highest wealth quantile.

### Supplementary Information

Supplementary Tables.

## Data Availability

The datasets generated and/or analyzed during the current study are available in the Measure DHS website https://dhsprogram.com/data/available-datasets.cfm.

## Abbreviations

BMI: Body mass index
CI: Confidence interval
EDHS: Ethiopian Demographic and Health Survey
NCDs: Non-communicable diseases
WHO: World Health Organization
