# Supplementary material for: Sustained underweight in rural areas and emergence of overweight in urban Ethiopian women: a multivariate analysis of EDHS data 2000–2016
Source: Sci Rep. 2024 Jul 19;14:16668. doi: 10.1038/s41598-024-66409-y (PMC11271595; doi:10.1038/s41598-024-66409-y)
Supplement: Supplementary file 1 — Supplementary Tables. [file 41598_2024_66409_MOESM1_ESM.pdf]

Sustained Underweight in Rural Areas and Emergence of Overweight in Urban Ethiopian Women: A Multivariate Analysis of EDHS Data 2000-2016

Amare Abera Tareke[1, 2*], Addis Alem[1], Wondwossen Debebe[1], Taddese Alemu Zerfu[3,4]

[1]Department of Biomedical Sciences, College of Medicine and Health Sciences, Wollo University, Dessie, Ethiopia

[2]Department of Public Health, College of Medicine and Health Sciences, Wollo University, Dessie, Ethiopia

[3]International Food Policy Research Institute (IFPRI), Addis Ababa, Ethiopia

[4]College of Medicine and Health Sciences, Dilla University, Dilla, Ethiopia

Correspondence to:

Amare Abera Tareke

Email; amareabera8@gmail.com

Phone; +251954596329

College of Medicine and Health Sciences, Wollo University, Dessie, Ethiopia

Supplementary Materials

Supplementary table 1: Trends in obesity

	2000	2005	2011	2016
**Obese**	163(1.3%)	89(1.7%)	353(2.6%)	493(3.9%)
**Others**	12273(98.7%)	5279(98.3%)	13011(97.4%)	12156(96.1%)

Supplementary table 2: Urban rural trends in Obesity

	2000	2005	2011	2016
**Urban**	113(4.57%)	74(6.94%)	257(7.53%)	373(12.6%)
**Rural**	50(0.5%)	15(0.34%)	96(0.96%)	120(1.2%)

Supplementary table 3: Mean BMI (Kg/m2) based on wealth index

	2005	2011	2016
**Poorest**	19.52	19.10	19.39
**Poorer**	19.53	19.47	19.94
**Middle**	19.55	19.51	19.95
**Rich**	19.79	19.93	20.38
**Richest**	21.47	21.78	22.48

Supplementary table 4: The percentage point differences in underweight and overweight Ethiopian women 2011 to 2016.

	Underweight			Overweight		
	2016	2011	Difference	2016	2011	Difference
**Region**						
**Tigray**	35.24	41.00	-5.76	5.96	3.31	2.65
**Afar**	40.44	43.86	-3.42	8.05	5.00	3.05
**Amhara**	23.64	30.49	-6.85	3.48	3.73	-0.25
**Oromia**	25.52	27.73	-2.21	7.63	5.04	2.59
**Somali**	31.67	34.07	-2.4	15.02	15.35	-0.33
**Ben-Gumz**	20.75	28.90	-8.15	7.68	3.14	4.54
**SNNP**	15.76	21.34	-5.58	5.86	5.80	0.06
**Gambela**	31.43	29.76	1.67	8.72	7.64	1.08
**Harari**	21.49	22.67	-1.18	20.49	14.40	6.09
**Addis Aba**	13.80	14.79	-0.99	29.42	19.97	9.45
**Dire Dawa**	22.94	25.78	-2.84	21.40	19.30	2.1
**Sex of household head**						
**Male**	23.67	29.00	-5.33	6.73	5.42	1.31
**Female**	21.79	24.02	-2.23	10.83	6.95	3.88
**Age in 5-year groups**						
**15-19**	29.42	36.27	-6.85	3.66	2.67	0.99
**20-24**	21.15	20.05	1.1	5.58	5.21	0.37
**25-29**	18.90	23.88	-4.98	8.10	5.28	2.82
**30-34**	19.92	24.05	-4.13	10.75	8.18	2.57

**35-39**	22.60	25.69	-3.09	10.72	7.73	2.99
**40-44**	22.68	26.83	-4.15	11.86	10.89	0.97
**45-49**	25.33	31.42	-6.09	9.45	7.46	1.99
**Highest educational level of the respondent**						
**No education**	24.18	27.46	-3.28	4.85	3.97	0.88
**Primary**	24.54	31.00	-6.46	7.42	5.46	1.96
**Secondary**	16.99	20.66	-3.67	12.63	12.64	-0.01
**Higher**	19.94	14.51	5.43	22.94	16.53	6.41
**Current contraceptive method**						
**No method**	24.91	29.72	-4.81	7.25	4.91	2.34
**Any method**	18.95	20.84	-1.89	9.09	8.90	0.19
**Type of place of residence**						
**Urban**	15.24	20.38	-5.14	21.25	14.93	6.32
**Rural**	25.62	30.24	-4.62	3.66	2.68	0.98
**Number of household members**						
**<5**	22.13	25.11	-2.98	8.85	6.75	2.1
**>=5**	23.77	29.00	-5.23	7.20	5.35	1.85
**Number of children 5 and under in household**						
**<=1**	23.02	27.17	-4.15	8.48	6.83	1.65
**>1**	23.78	29.35	-5.57	5.30	2.78	2.52
**Total children ever born**						
**<=2**	23.12	28.60	-5.48	7.89	5.81	2.08
**>2**	23.26	26.51	-3.25	7.65	5.81	1.84
**Births in last five years**						
**<=1**	23.22	28.12	-4.9	8.35	6.37	1.98
**>1**	22.98	25.47	-2.49	4.38	2.68	1.7
**Religion**						
**Orthodox**	22.84	28.77	-5.39	8.97	6.35	2.62
**Muslim**	27.35	30.28	-2.93	6.55	5.36	1.19
**Others**	19.17	22.76	-3.59	7.06	5.18	1.88
**Currently/formerly/never in union**						
**Never in union**	25.53	32.00	-6.47	6.02	4.19	1.83
**Currently**	22.09	26.09	-4	8.03	6.33	1.7
**Formerly**	22.61	23.88	-1.27	11.48	7.74	3.74
**Respondent currently working**						
**No**	24.99	29.32	-4.33	5.52	4.69	0.83
**Yes**	19.87	25.27	-5.4	11.95	7.74	4.21
**Type of earnings from respondent's work**						
**Not paid**	22.53	32.16	-9.63	4.96	2.51	2.45
**Cash only**	17.67	21.80	-4.13	15.35	10.36	4.99
**Cash & in-kind**	18.60	29.28	-10.68	8.65	4.63	4.02
**In-kind only**	25.92	32.74	-6.82	5.29	1.28	4.01
**Wealth index**						
**Poorest**	29.35	33.70	-4.35	2.53	1.90	0.63
**Poorer**	24.96	31.98	-7.02	2.67	2.37	0.3
**Middle**	25.32	28.32	-3	2.63	1.81	0.82
**Richer**	24.15	29.65	-5.5	4.96	2.62	2.34
**Richest**	16.48	19.32	-2.84	19.45	15.56	3.89

Supplementary table 5: The percentage point differences in underweight and overweight Ethiopian women 2005 to 2016.

	Underweight			Overweight		
	2016	2005	Difference	2016	2005	Difference
**Region**						
**Tigray**	35.24	38.31	-3.07	5.96	1.52	4.44
**Afar**	40.44	33.34	7.1	8.05	3.93	4.12
**Amhara**	23.64	28.59	-4.95	3.48	2.51	0.97
**Oromia**	25.52	25.27	0.25	7.63	4.56	3.07
**Somali**	31.67	37.76	-6.09	15.02	10.64	4.38
**Ben-Gumz**	20.75	34.56	-13.81	7.68	2.17	5.51
**SNNP**	15.76	26.89	-11.13	5.86	3.06	2.8
**Gambela**	31.43	38.15	-6.72	8.72	2.18	6.54
**Harari**	21.49	21.17	0.32	20.49	9.79	10.7
**Addis Aba**	13.80	15.46	-1.66	29.42	18.16	11.26
**Dire Dawa**	22.94	24.07	-1.13	21.40	14.40	7.0
**Sex of household head**						
**Male**	23.67	27.49	-3.82	6.73	3.85	2.88
**Female**	21.79	26.87	-5.08	10.83	6.82	4.01
**Age in 5-year groups**						
**15-19**	29.42	33.12	-3.7	3.66	3.66	0
**20-24**	21.15	20.08	1.07	5.58	2.66	2.92
**25-29**	18.90	22.97	-4.07	8.10	4.37	3.73
**30-34**	19.92	23.40	-3.48	10.75	6.87	3.88
**35-39**	22.60	27.50	-4.9	10.72	5.33	5.39
**40-44**	22.68	28.86	-6.18	11.86	5.75	6.11
**45-49**	25.33	33.69	-8.36	9.45	6.02	3.43
**Highest educational level of the respondent**						
**No education**	24.18	28.36	-4.18	4.85	2.85	2
**Primary**	24.54	29.21	-4.67	7.42	3.48	3.94
**Secondary**	16.99	19.28	-2.29	12.63	14.18	-1.55
**Higher**	19.94	18.42	1.52	22.94	17.29	5.65
**Current contraceptive method**						
**No method**	24.91	28.89	-3.98	7.25	4.13	3.12
**Any method**	18.95	16.26	2.69	9.09	7.66	1.43
**Type of place of residence**						
**Urban**	15.24	19.24	-4	21.25	14.65	6.6
**Rural**	25.62	29.35	-3.73	3.66	2.05	1.61
**Number of household members**						

**<5**	22.13	24.55	-2.42	8.85	6.01	2.84
**>=5**	23.77	28.57	-4.8	7.20	3.93	3.27
**Number of children 5 and under in household**						
**<=1**	23.02	27.98	-4.96	8.48	5.49	2.99
**>1**	23.78	25.72	-1.94	5.30	2.21	3.09
**Total children ever born**						
**<=2**	23.12	27.07	-3.95	7.89	5.13	2.76
**>2**	23.26	27.66	-4.4	7.65	3.89	3.76
**Births in last five years**						
**<=1**	23.22	28.33	-5.11	8.35	5.08	3.27
**>1**	22.98	23.03	-0.05	4.38	2.30	2.08
**Religion**						
**Orthodox**	22.84	26.12	-3.28	8.97	26.12	-17.15
**Muslim**	27.35	30.51	-3.16	6.55	30.51	-23.96
**Others**	19.17	25.96	-6.79	7.06	25.96	-18.9
**Currently/formerly/never in union**						
**Never in union**	25.53	28.90	-3.37	6.02	4.78	1.24
**Currently**	22.09	26.21	-4.12	8.03	4.23	3.8
**Formerly**	22.61	28.57	-5.96	11.48	5.55	5.93
**Respondent currently working**						
**No**	24.99	28.39	-3.4	5.52	3.56	1.96
**Yes**	19.87	25.08	-5.21	11.95	6.75	5.2
**Type of earnings from respondent's work**						
**Not paid**	22.53	24.84	-2.31	4.96	2.33	2.63
**Cash only**	17.67	22.09	-4.42	15.35	10.72	4.63
**Cash & in-kind**	18.60	39.59	-20.99	8.65	6.60	2.05
**In-kind only**	25.92	36.15	-10.23	5.29	0.12	5.17
**Poorest**	29.35	31.19	-1.84	2.53	1.75	0.78
**Poor**	24.96	30.77	-5.81	2.67	2.88	-0.21
**Middle**	25.32	30.95	-5.63	2.63	1.80	0.83
**Richer**	24.15	27.78	-3.63	4.96	1.74	3.22
**Richest**	16.48	19.93	-3.45	19.45	11.11	8.34

Supplementary table 6: multivariate decomposition analysis of percentage changes in underweight among Ethiopian women 2011-2016

**Variables**	**Difference due to characteristics (E)**		**Difference due to coefficients (C)**	
	Coefficient (95% CI)	Pct	Coefficient (95% CI)	Pct
**Region**				
**Tigray**				
**Afar**	0.00000(-0.00003, 0.00004)	0.01	0.00011(-0.00044, 0.00067)	o.25
**Amhara**	-0.00316(-0.00409, -0.00224) **	7.00	0.00606(-0.00634, 0.01845)	13.40
**Oromia**	-0.00078(-0.00106, -0.00050) **	-1.73	-0.00187(-0.02050, 0.01676)	-1.24
**Somali**	0.00079(0.00034, 0.00124) **	1.75	0.00055(-0.00058, 0.00169)	1.23
**Ben-Gumz**	-0.00007(-0.00010, -0.00005) **	-0.16	0.00060(0.00000, 0.00120) *	1.34

**SNNP**	0.00437(0.00338, 0.00537) **	9.68	0.00984(-0.00166, 0.02135)	21.78
**Gambela**	-0.00002(-0.00009, 0.00005)	-0.05	-0.00023(-0.00052, 0.00005)	-0.51
**Harari**	-0.00008(-0.00011, -0.00004) **	0.-17	-0.00003(-0.00023, 0.00017)	-0.07
**Addis Aba**	0.00071(0.00046, 0.00096) **	0.157	-0.00272(-0.00651, 0.00108)	-6.01
**Dire Dawa**	0.00011(0.00004, 0.00018) **	0.25	-0.00000(-0.00027, 0.00026)	-0.01
**Sex of household head**				
**Male**				
**Female**	0.00001(-0.00001, 0.00002)	0.01	-0.00661(-0.01766, 0.00445)	-14.62
**Age in 5-year groups**				
**15-19**				
**20-24**	-0.00028(-0.00045, -0.00010) **	-0.62	-0.00513(-0.01430, 0.00404)	-11.35
**25-29**	0.00006(0.00003, 0.00008) **	0.12	0.00850(-0.00232, 0.01932)	18.81
**30-34**	0.00264(0.00115, 0.00412) **	5.83	0.00756(-0.00091, 0.01603)	16.72
**35-39**	0.00079(0.00019, 0.00140) **	1.76	0.00587(-0.00291, 0.01465)	12.99
**40-44**	0.00067(0.00017, 0.00118) **	1.49	0.00551(-0.00138, 0.01240)	12.19
**45-49**	-0.00057(0.00017, 0.00118) *	-1.27	0.00761(0.00009, 0.01513) *	16.84
**Highest educational level of the respondent**				
**No education**				
**Primary**	0.00015(-0.00103, 0.00133)	0.34	0.01068(-0.00595, 0.02731)	23.63
**Secondary**	0.00220(0.00022, 0.00417) *	4.86	0.00263(-0.00254, 0.00780)	5.82
**Higher**	-0.00071(-0.00141, -0.00002)	-1.58	-0.00539(-0.00975, -0.00103) *	-11.94
**Current contraceptive method**				
**No method**				
**Any method**	0.00199(0.00012, 0.00386) *	4.40	-0.00254(-0.01240, 0.00732)	-5.62
**Type of place of residence**				
**Urban**				
**Rural**	-0.00173-0.00286, -0.00059) **	-3.82	-0.05195(-0.10738, 0.00349)	-114.94
**Number of household members**				
**<5**				
**>=5**	-0.00049(-0.00118, 0.00019)	-1.09	0.00753(-0.01885, 0.03391)	16.67
**Number of children 5 and under in household**				
**<=1**				
**>1**	-0.00036(-0.00157, 0.00084)	-0.81	0.00939(-0.00300, 0.02177)	20.77
**Total children ever born**				
**<=2**				
**>2**	-0.00080(-0.00156, -0.00004) *	-1.77	-0.02481(-0.04959, -0.00003)	-54.90
**Births in last five years**				
**<=1**				
**>1**	-0.00001(-0.00047, 0.00046)	-0.01	-0.00332(-0.012580.00593)	-7.36
**Religion**				
**Orthodox**				
**Muslim**	-0.00085(-0.00175, 0.00006)	-1.88	-0.00594(0.01774, 0.00585)	-13.15
**Others**	-0.00032(-0.00105, 0.00041)	-0.71	-0.00971(-0.02310, 0.00367)	-21.49
**Currently/formerly/never in union**				
**Never in union**				
**Currently**	0.00099(-0.00044, 0.00241)	2.18	-0.00667(-0.03923, 0.02589)	-14,77
**Formerly**	-0.00040(-0.00119, 0.00040)	-0.88	-0.00451(-0.01270, 0.00368)	-9.98
**Respondent currently working**				
**No**				
**Yes**	0.00090(-0.00196, 0.00016)	-2.00	0.01018(-0.00353, 0.02389)	22.52
**Wealth index**				
**Poorest**				
**Poor**	-0.00028(-0.00064, 0.00008)	-0.62	0.00432(-0.00479, 0.01342)	9.55

**Middle**	0.00017(-0.00021, 0.00055)	0.38	-0.00372(-0.01298, 0.00554)	-8.22
**Richer**	0.00014(-0.00008, 0.00036)	0.31	-0.00004(-0.01034, 0.01026)	-0.09
**Richest**	0.00045(0.00006, 0.00083) *	0.99	-0.00952(-0.03014, 0.01111)	-21.06
**Overall**	0.00440(0.00039, 0.00841) **	9.74	0.04079(0.02476, 0.05682) **	90.6

*p value ≤ 0.05, **p value ≤ 0.01, CI; confidence interval, pct; percentage

Supplementary table 7: multivariate decomposition analysis of percentage changes in overweight among Ethiopian women 2011-2016

**Variables**	**Difference due to characteristics (E)**		**Difference due to coefficients (C)**	
	Coefficient (95% CI)	Pct	Coefficient (95% CI)	Pct
**Region**				
**Tigray**				
**Afar**	0.00002(0.00000, 0.00004) *	-0.10	-0.00005(-0.00041, 0.00032)	0.24
**Amhara**	-0.00057(-0.00114, -0.00001) *	2.89	0.01320(-0.00161, 0.02802)	-66.33
**Oromia**	0.00018(0.00004, 0.00031) **	-0.90	0.00503(-0.00736, 0.01741)	-25.25
**Somali**	-0.00084(-0.00110, -0.00058) **	4.21	-0.00013(-0.00083, 0.00057)	0.66
**Ben-Gumz**	0.00001(0.00001, 0.00002) **	-0.08	-0.00029(-0.00078, 0.00020)	1.44
**SNNP**	-0.00047(-0.00088, -0.00007) *	2.38	0.00560(-0.00243, 0.01363)	-28.14
**Gambela**	0.00001(-0.00002, 0.00004)	-0.04	0.00021(-0.00003, 0.00044)	-1.03
**Harari**	0.00003(0.00002, 0.00005) **	-0.16	-0.00000(-0.00010, 0.00010)	0.01
**Addis Aba**	-0.00022(-0.00033, -0.00012) **	1.13	0.00033(-0.00144, 0.00210)	-1.67
**Dire Dawa**	-0.00006(-0.00009, -0.00003) **	0.32	0.00008(-0.00007, 0.00023)	0.38
**Sex of household head**				
**Male**				
**Female**	-0.00000(-0.00001, 0.00000)	0.01	-0.00537(-0.01224, 0.0015)	26.97
**Age in 5-year groups**				
**15-19**				
**20-24**	0.00005(-0.00003, 0.00014)	0.27	0.00195(-0.00425, 0.00814)	-9.77
**25-29**	-0.00002(-0.00004, -0.00001) **	0.11	-0.00094(-0.00789, 0.00601)	4.71
**30-34**	-0.00173(-0.00246, -0.00100) **	8.68	-0.0003(-0.00516, 0.00454)	1.56
**35-39**	-0.00067(-0.00096, -0.00038)	3.38	0.00070(-0.00398, 0.00538)	-3.52
**40-44**	-0.00060(-0.00085, -0.00035)	3.01	0.00254(-0.00108, 0.00616)	-12.74
**45-49**	0.00054(0.00027, 0.00082)	-2.73	0.00235(-0.00174, 0.00644)	11.80
**Highest educational level of the respondent**				
**No education**				
**Primary**	0.00080(0.00022, 0.00138) **	-4.00	-0.00428(-0.01534, 0.00678)	21.52
**Secondary**	-0.00130(-0.00223, -0.00036) **	6.51	-0.00059(-0.00307, 0.00190)	2.94
**Higher**	-0.00040(-0.00066, -0.00013) **	1.99	-0.00084(-0.00276, 0.00108)	4.23
**Current contraceptive method**				
**No method**				

**Any method**	-0.00017(-0.00093, 0.00059)	0.85	0.00252(-0.00292, 0.00797)	-12.68
**Type of place of residence**				
**Urban**				
**Rural**	0.0008190.00045, 0.001170**	-4.04	0.01869 (-0.01108, 0.04847)	-93.92
**Number of household members**				
**<5**				
**>=5**	0.00024(-0.00018, 0.00066)	1.39	-0.00099(-0.01547, 0.01348)	5.00
**Number of children 5 and under in household**				
**<=1**				
**>1**	0.00009(-0.00020, 0.00039)	-0.47	-0.00923(-0.01908, 0.00063)	46.36
**Total children ever born**				
**<=2**				
**>2**	0.00009(-0.00020, 0.00039)	-7.36	-0.00695(-0.02038, 0.00648)	34.91
**Births in last five years**				
**<=1**				
**>1**	-0.00025(-0.00047, -0.00002) *	1.25	0.00301(-0.00341, 0.00943)	-15.91
**Religion**				
**Orthodox**				
**Muslim**	0.00013(-0.00026, 0.00053)	-0.66	0.00322(-0.00388, 0.01032)	-16.18
**Others**	0.00015(-0.00013, 0.00043)	0.75	0.00018(-0.00662, 0.00698)	-0.91
**Currently/formerly/never in union**				
**Never in union**				
**Currently**	-0.00076(-0.00142, -0.00010) *	3.81	-0.00510(-0.02361, 0.01341)	25.61
**Formerly**	0.00040(0.00005, 0.00075) *	-2.00	-0.00024(-0.00486, 0.00438)	1.20
**Respondent currently working**				
**No**				
**Yes**	0.00024(-0.00018, 0.00066)	-1.20	-0.00362(-0.01152, 0.00428)	18.18
**Wealth index**				
**Po0rest**				
**poor**	0.00017(-0.00009, 0.00044)	0.87	-0.00097(-0.01007, 0.00814)	4.85
**Middle**	-0.00016(-0.00043, 0.00011)	0.80	-0.00415(-0.01409, 0.00579)	20.86
**Richer**	-0.00025(-0.00038, -0.00012) **	1.27	-0.00777(-0.01938, 0.00385)	39.02
**Richest**	-0.00059(-0.00079, -0.00038) **	2.95	0.00412(-0.00763, 0.01586)	-20.68
**Overall**	-0.00493(-0.00675, -0.00310) **	24.75	-0.01498(-0.02308, -0.00688)	75.25

*p value ≤ 0.05, **p value ≤ 0.01, CI; confidence interval, pct; percentage

Supplementary table 8: multivariate decomposition analysis of percentage changes in underweight among Ethiopian women 2005-2016

**Variables**	**Difference due to characteristics (E)**		**Difference due to coefficients (C)**	
	Coefficient (95% CI)	Pct	Coefficient (95% CI)	pct
**Region**				
**Tigray**				
**Afar**	0.00001(-0.00011, 0.00013)	0.02	-0.00069(-0.00160, 0.00023)	-1.67
**Amhara**	0.00060(0.00042, 0.00078) **	1.46	0.00484(-0.00899, 0.01868)	11.77
**Oromia**	0.00027(0.00017. 0.00038) **	0.67	-0.00386(-0.02596, 0.01825)	-9.37
**Somali**	-0.00073(-0.00116, -0.00030) **	-1.78	0.00191(-0.00106, 0.00488)	4.64
**Ben-Gumz**	0.00013(0.00009, 0.00017) **	0.31	0.00098(0.00031, 0.00165) **	2.38

**SNNP**	-0.00212(-0.00266, -0.00158) **	-5.14	0.02222(0.00695, 0.03748) **	54.00
**Gambela**	-0.00001(-0.00003, 0.00001) **	-0.02	0.00003(-0.00024, 0.00031)	0.08
**Harari**	-0.00008(-0.00012, -0.00004) **	-0.19	-0.00002(-0.00028, 0.00024) **	-0.05
**Addis Aba**	0.00062(0.00037, 0.00087) **	1.51	-0.00127(-0.00602, 0.00347) **	-3.10
**Dire Dawa**	0.00002(0.00001, 0.00003) **	0.04	-0.00005(-0.00050, 0.00040)	-0.13
**Sex of household head**				
**Male**				
**Female**	0.00025(-0.00025, 0.00075)	0.84	0.00359(-0.00788, 0.01506)	8.72
**Age in 5-year groups**				
**15-19**				
**20-24**	-0.00034(-0.00056, -0.00013) *	0.83	-0.00462(-0.01497, 0.00573)	-11.23
**25-29**	0.00168(0.00076, 0.00260) **	4.08	0.00400(-0.00685, 0.01485)	9.73
**30-34**	0.00249(0.00101, 0.00396) **	6.04	0.00283(-0.00666, 0.01231)	6.87
**35-39**	0.00065(0.00014, 0.00117) *	1.58	0.00404(-0.00592, 0.01400)	9.83
**40-44**	-0.00014(-0.00024, -0.00003) *	-0.33	0.00404(-0.00454, 0.01262)	9.82
**45-49**	-0.00096(-0.00194, 0.00002) *	-2.34	0.00532(-0.00372, 0.01436)	12.93
**Highest educational level of the respondent**				
**No education**				
**Primary**	-0.00042(-0.00362, 0.00278)	-1.02	0.00417(-0.00759, 0.01592)	10.13
**Secondary**	0.00012(-0.00000, 0.00025)	0.30	0.00427(-0.00438, 0.01292)	10.38
**Higher**	-0.00269(-0.00517, -0.00021) **	-654	-0.00085(-0.00296,0.00127)	2.06
**Current contraceptive method**				
**No method**				
**Any method**			-0.00739(-0.01535, 0.00058)	-17.95
**Type of place of residence**				
**Urban**				
**Rural**	0.00271(0.00107, 0.00435) **	-6.59	-0.04580(-0.11141, 0.01982)	-111.32
**Number of household members**				
**<5**				
**>=5**	-0.00093(-0.00226, 0.00039)	-2.27	0.03581(0.00404, 0.06757) *	87.03
**Number of children 5 and under in household**				
**<=1**				
**>1**	-0.00066(-0.00284, 0.00151)	-1.61	0.00094(-0.01456, 0.01644)	2.29
**Total children ever born**				
**<=2**				
**>2**	0.00065(0.00003, 0.00127) *	1.59	-0.01656(-0.04731, 0.01418)	-40.26
**Births in last five years**				
**<=1**				
**>1**	-0.00002(-0.00188, 0.00184)	-0.05	-0.00867(-0.02168, 0.00434)	21.07
**Religion**				
**Orthodox**				
**Muslim**	-0.00002(-0.00004, 0.00000)	-0.05	0.00033(-0.01459, 0.01525)	0.80
**Others**	-0.00074(-0.00244, 0.00096)	-1.80	-0.00265(-0.01595, 0.01065)	-6.44
**Currently/formerly/never in union**				
**Never in union**				
**Currently**	0.00076(-0.00033, 0.00185)	1.84	0.02064(-0.01597, 0.05725)	50.16
**Formerly**	-0.00051(-0.00151, 0.00050	-1.23	0.00480(-0.00467, 0.01427)	11.66
**Respondent currently working**				
**No**				
**Yes**	0.00075(-0.00010, 0.00159)	1.82	0.00230(-0.01044, 0.01505)	5.60
**Wealth index**				
**Poorest**				
**Poorer**	-0.00050(-0.00114, 0.000150)	-1.21	0.00891(-0.00238, 0.02019)	21.65

**Middle**	0.00018(-0.00022, 0.00059)	0.45	0.00689(-0.00396, 0.01775)	16.75
**Richer**	0.00058(-0.00032, 0.00149)	1.42	0.00373(-0.00739, 0.01485)	9.06
**Richest**	0.00008(0.00001, 0.00016) *	0.20	0.00315(-0.01902, 0.02532)	7.65
**Overall**	0.00649(-0.00085, 0.01383)	15.77	0.03465(0.01451, 0.05480) **	84.23

*p value ≤ 0.05, **p value ≤ 0.01, CI; confidence interval, pct; percentage

Supplementary table 9: multivariate decomposition analysis of percentage changes in overweight among Ethiopian women 2005-2016

**Variables**	**Difference due to characteristics (E)**		**Difference due to coefficients (C)**	
	Coefficient (95% CI)	Pct	Coefficient (95% CI)	Pct
**Region**				
**Tigray**				
**Afar**	0.00008(0.00001, 0.00015) *	-0.26	0.00037(-0.00039, 0.00113)	-1.13
**Amhara**	0.00013(-0.00000, 0.00027)	-0.41	0.01795(-0.00014, 0.03605)	-54.65
**Oromia**	-0.00008(-0.00013, -0.00002) **	0.23	0.01494(-0.00538, 0.03526)	-45.48
**Somali**	0.00096(0.00067, 0.00124) **	-2.91	0.00080(-0.00147, 0.00307)	-2.44
**Ben-Gumz**	-0.00003(-0.00005, -0.00001) **	0.10	0.00007(-0.00053, 0.00067)	-0.21
**SNNP**	0.00028(0.00003, 0.00054) *	-0.86	0.01116(-0.00280, 0.02512)	-33.97
**Gambela**	.00000(-0.00001, 0.00002)	-0.01	0.00011(-0.00014, 0.00037)	-0.35
**Harari**	0.00004(0.00002, 0.00006) **	-0.12	0.00001(-0.00017, 0.00018)	0.03
**Addis Aba**	-0.00024(-0.00035, -0.00014) **	0.74	0.00230(-0.00104, 0.00565)	7.01
**Dire Dawa**	0.00001(-0.00002, -0.00001) **	0.03	0.00018(-0.00015), 0.00050)	-0.54
**Sex of household head**				
**Male**				
**Female**	-0.00008(-0.00033, 0.00016)	0.25	-0.00063(-0.00744, 0.00618)	1.92
**Age in 5-year groups**				
**15-19**				
**20-24**	0.00008(-0.00005, 0.00021)	-0.25	-0.00894(-0.01818, 0.00030)	27.21
**25-29**	-0.000849-0.00134, -0.00034) *	2.56	-0.00648(-0.01443, 0.00147)	19.72
**30-34**	-0.00201(-0.00279, -0.00122) **	6.11	-0.00369(-0.00994, 0.00256)	11.24
**35-39**	-0.00068(-0.00095, -0.00041) **	2.07	-0.00464(-0.01148, 0.00220)	14.12
**40-44**	0.0001590.00009, 0.00020) **	-0.45	-0.00440(-0.01054, 0.00173)	13.41
**45-49**	0.00113(0.00062, 0.00163) **	-3.43	-0.00171(-0.00739, 0.00396)	5.21
**Highest educational level of the respondent**				
**No education**				
**Primary**	-0.00269(-0.00441, -0.00097) **	8.19	0.00451(-0.01478, 0.00575)	13.74
**Secondary**	-0.00009(-0.00014, -0.00004) **	0.27	0.00136(-0.00328, 0.00600)	-4.14
**Higher**	-0.00184(-0.00292, -0.00075) **	5.60	-0.00010(-0.00088, 0.00069)	0.29
**Current contraceptive method**				
**No method**				
**Any method**	-0.00051(-0.00277, 0.00176)	1.54	0.00048(-0.00312, 0.00408)	-1.45
**Type of place of residence**				
**Urban**				
**Rural**	-0.00156(-0.00238, -0.00074) **	4.74	-0.02614(-0.06258, 0.01029)	79.58
**Number of household members**				
**<5**				
**>=5**	0.00064(-0.00004, 0.00133)	-1.96	-0.01402(-0.03414, 0.00611)	42.67
**Number of children 5 and under in household**				
**<=1**				
**>1**	0.00062(-0.00066, 0.00190)	-1.88	-0.01048(-0.02372, 0.00276)	31.90
**Total children ever born**				
**<=2**				

**>2**	-0.00009(-0.00039, 0.00020)	0.29	-0.00575(-0.02278, 0.01128)	17.49
**Births in last five years**				
**<=1**				
**>1**	-0.00123(-0.00232, -0.00014) *	3.74	0.00594(-0.0050, 0.01688)	-18.07
**Religion**				
**Orthodox**				
**Muslim**	0.00000(-0.00001, 0.00002)	-0.01	0.00507(-0.00447, 0.01461)	-15.43
**Others**	0.00042(-0.00038, 0.00122)	-1.29	0.00126(-0.00655, 0.00907)	3.84
**Currently/formerly/never in union**				
**Never in union**				
**Currently**	-0.00072(-0.00136, -0.00007) *	2.19	-0.00446(-0.02720, 0.01828)	13.58
**Formerly**	0.00063(0.00006, 0.00119) *	-1.91	-0.00309(-0.00950, 0.00333)	19.39
**Respondent currently working**				
**No**				
**Yes**	-0.00024(-0.00068, 0.00019)	0.74	0.00516(-0.00293, 0.01326)	-15.71
**Wealth index**				
**Po0rest**				
**poor**	0.00038(-0.00022, 0.00097)	-1.14	0.00416(-0.00779, 0.01611)	-12.67
**Middle**	-0.00021(-0.00057, 0.00016)	0.63	-0.00017(-0.01214, 0.01180)	0.52
**Rich**	-0.00130(-0.00205, -0.00054) **	3.94	-0.00857(-0.02153, 0.00439)	26.09
**Richest**	-0.00014(-0.00019 -0.00008) **	0.41	-0.01454(-0.03611, 0.00702)	44.27
**Overall**	-0.00922(-0.01271, -0.00572) **	28.06	-0.02364, -0.03263, -0.01464) **	71.94

*p value ≤ 0.05, **p value ≤ 0.01, CI; confidence interval, pct; percentage
